# Proteomic Approach during the Induction of Somatic Embryogenesis in *Coffea canephora*

**DOI:** 10.3390/plants12244095

**Published:** 2023-12-07

**Authors:** Ana Odetth Quintana-Escobar, Esaú Bojórquez-Velázquez, Eliel Ruiz-May, Víctor Manuel Loyola-Vargas

**Affiliations:** 1 Unidad de Bioquímica y Biología Molecular de Plantas, Centro de Investigación Científica de Yucatán, Calle 43, No. 130 x 32 y 34, Mérida CP 97205, Yucatán, Mexico; ana.quintana@estudiantes.cicy.mx; 2 Red de Estudios Moleculares Avanzados, Clúster Científico y Tecnológico BioMimic®, Instituto de Ecología A.C. (INECOL), Carretera Antigua a Coatepec No. 351, Congregación el Haya, Xalapa CP 91070, Veracruz, Mexico; esau.bojorquez@inecol.mx (E.B.-V.); eliel.ruiz@inecol.mx (E.R.-M.)

**Keywords:** *Coffea canephora*, mass spectrometry, plant tissue culture, somatic embryogenesis

## Abstract

Plant growth regulators (PGR) are essential for somatic embryogenesis (SE) in different species, and *Coffea canephora* is no exception. In our study model, previously, we have been able to elucidate the participation of various genes involved in SE by using different strategies; however, until now, we have not used a proteomic approach. This research seeks to contribute to understanding the primary cellular pathways involved in developing SE in *C. canephora*. The process of our model consists of two stages: (1) preconditioning in MS medium with auxin (NAA) and cytokinin (KIN), and (2) induction in Yasuda liquid medium added with cytokinin (BA). Therefore, in this study, we analyzed different days of the SE induction process using shotgun label-free proteomics. An amount of 1630 proteins was found among different sampling days of the process, of which the majority were accumulated during the induction stage. We found that some of the most enriched pathways during this process were the biosynthesis of amino acids and secondary metabolites. Eighteen proteins were found related to auxin homeostasis and two to cytokinin metabolism, such as ABC, BIG, ILR, LOG, and ARR. Ten proteins and transcription factors related to SE were also identified, like SERK1, SKP1, nuclear transcription factor Y, MADS-box, and calreticulin, and 19 related to other processes of plant development, among which the 14-3-3 and PP2A proteins stand out. This is the first report on the proteomic approach to elucidate the mechanisms that operate during the induction of SE in *C. canephora*. So, our findings provide the groundwork for future, more in-depth research. Data are available via ProteomeXchange Consortium via the PRIDE partner repository with the dataset identifier PXD047172.

## 1. Introduction

Somatic embryogenesis (SE) is an effective biotechnological tool for studying the morpho-physiological, biochemical, and molecular processes that develop crops of interest, such as the coffee plant. Also, SE represents a viable alternative that allows the mass production of homogeneous plants, free of diseases and with desirable agronomic characteristics, in less time and space.

The genus *Coffea* comprises more than 127 species of evergreen woody trees with differences in size, morphology, and adaptation conditions. Among these species, *C. canephora* and *C. arabica* are the most cultivated worldwide [1] with a worldwide production of 40 and 60%, respectively [2]. Among them, *C. canephora* has a higher caffeine content and is more resistant to pests, diseases [3], and extreme climatic changes than *C. arabica*. Given the importance of this crop, various investigations have been developed related to the increase in mass propagation, its genetic improvement, and improvement in agronomic qualities such as productivity, grain quality, physicochemical processes, and resistance to biotic and abiotic factors, among others.

SE has currently been used to study cell differentiation in the *C. canephora* system, and it has been shown that plant growth regulators (PGRs) play a primary role in cell signaling and differentiation [4]. It has been found that during the SE process in *C. canephora*, the most important auxin (indole3-acetic acid/IAA) plays a fundamental role. It has become the object of study to elucidate the mechanisms involved in its biosynthesis, transport, signaling, accumulation, and homeostasis [5]. Other important but less studied regulators are cytokinins. Auxins and cytokinins are two of the most significant plant growth regulators, and they play a crucial role in all areas of plant growth and development [6]. These two PGRs are involved in cell division, elongation, differentiation, vascular and flower development, nutrient homeostasis, leaf expansion, and stress responses, among others [7,8].

Although transcriptomics broadened the panorama for the correct understanding of the first stages of the SE process by studying the genes involved in the embryogenic response [9,10,11,12,13], there is another area that has gained great scope in recent years: proteomics. Proteomics offers a closer approach to the state of the cell in a specific space and time [14,15,16].

Despite the valuable results obtained using transcriptomics regarding the genes directly involved in SE, the lack of correlation between the number of transcripts and the abundance of proteins may be a limiting factor. It is known that not all transcribed genes are indeed translated into their functional products. In addition, translation can be affected by many factors, which interfere with the interpretation of results obtained at a precise time [17]. Among the reasons that cause this lack of correlation are variations in mRNA stability, its translation, protein stability, changes in structure, activity, and function due to its cellular location, interaction with other molecules, or post-translational modifications [18]. The proteomic approaches are the best techniques to study global plant proteome on a large scale and with high throughput since they allow for the quantitative and qualitative analysis of proteins involved in somatic embryogenesis, providing a more precise biochemical state of cells and the changes that occur during their development. For the above, it is increasingly a requirement to complement the information of the transcripts with the final functional products of the genes: the proteins. Proteomics helps elucidate the biochemical and molecular processes necessary to carry out SE via the identification and/or quantification of proteins with differential abundances [19,20].

In spite of the increased use of proteomic strategies, more is needed to know about the study of *Coffea* spp. A few studies focus on the comparison of embryogenic versus non-embryogenic lines. In one of the first reported works on proteomics in *C. arabica*, three stages of somatic embryo development were evaluated via 2DE and mass spectrometry [21]. Specific proteins were identified at each stage. It was proposed that enolase and 11S storage globulin proteins could serve as molecular markers for embryo development and for the differentiation between embryogenic and non-embryogenic lines. Another work evaluated two embryogenic suspensions via mass spectrometry and shotgun [22]. The proteome was characterized, and proteins related to stress and energy production were identified. Although the analysis is very descriptive, it lays the groundwork for considering possible molecular markers for SE. Another investigation focused on the comparison, via 2DE, the extracellular proteome of embryogenic and non-embryogenic cell suspensions of *C. arabica* and *C. canephora* [23]. It was found that a larger population of proteins secreted into the medium of *C. canephora* compared to *C. arabica* and those proteins are secreted exclusively under embryogenic conditions. However, the identification of these proteins was not possible. So far, to our knowledge, there are no reports on the study of the proteome during the induction of somatic embryogenesis of *C. canephora*. Thus, this work aims to contribute to understanding the main cellular mechanisms involved during the induction of SE in *C. canephora* via a proteomic approach.

## 2. Results

**Figure 1:**
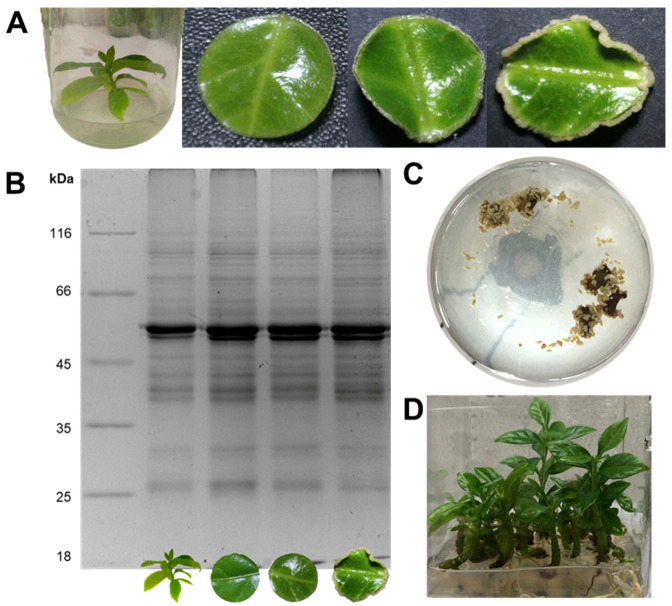
Somatic embryogenesis in *C. canephora***.** (**A**) From left to right: plantlet and leaf explant at 14 and 0 days before induction (dbi); leaf explant at 7 and 21 days after induction (dai). (**B**) 1D-SDS-PAGE visualization of the protein profiles from 14 dbi, 0 dbi, 7 dai, and 21 dai samples (10 µg protein). (**C**) Flask with somatic embryos released into the culture medium. (**D**) Somatic embryos germinated and converted to complete seedlings.

The induction of SE in *C. canephora* was carried out successfully. This process consisted of cultivating *C. canephora* seedlings for 14 days in a medium added with plant growth regulators (PGR) that favor the subsequent embryogenesis induction. After preconditioning, the circular explants were placed in a liquid induction medium with benzyladenine (BA) as the only PGR. At 7 day after induction, a slight change was observed in the periphery of the explants due to an increase in the size of the explants. By 21 day, the growth of proembryogenic structures was already evident throughout the periphery of the explants. Fourteen dbi (control), 0 dbi (seedlings at the end of 14 day in preconditioning medium), and 7 and 21 day after induction were selected as sampling days (Figure 1A). Day 14 dbi was selected as a control to make comparisons in subsequent analyses because the seedlings were under maintenance in a culture medium without PGR.

The quality of the extracted proteins for each sampling day was verified on 1D-SDS-PAGE gels (Figure 1B), in which no difference was visually observed between the different sampling points. It is worth mentioning that the development process of the somatic embryos was carried out to the end to verify the protocol’s effectiveness. At 56 d, embryos released into the culture medium could already be observed at different stages of development (Figure 1C). These embryos were collected and placed in a semisolid culture medium without PGR for germination and conversion to complete seedlings (Figure 1D), which can be used to start a new SE process.

**Figure 2:**
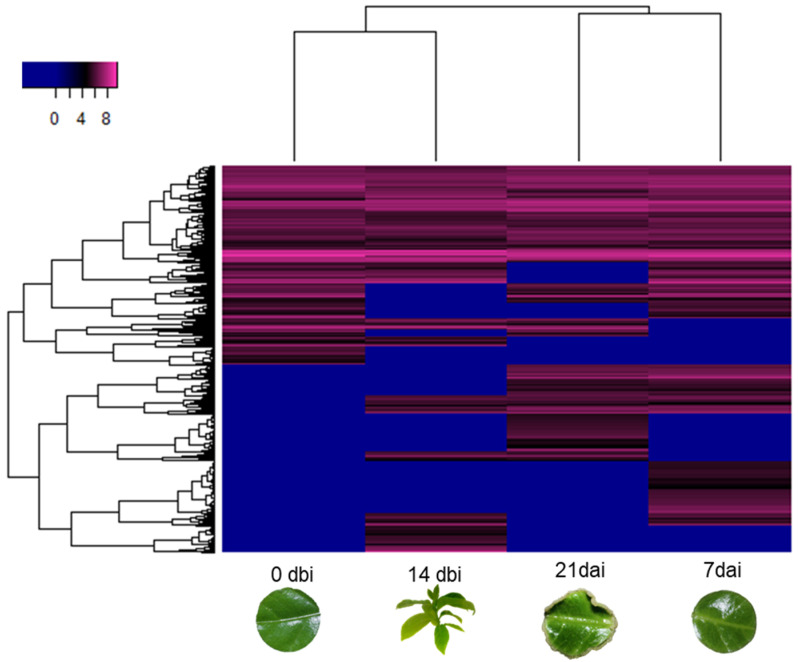
Proteomic distribution of somatic embryogenesis induction in *C. canephora*. Heatmap depicting the abundance of the 1630 proteins found among different sampling days of the process.

After proteomic analysis via LC-MS/MS, 1630 proteins accumulated on the different sampling days were identified. When performing the heatmap (Figure 2), two different clusters were observed: in the first cluster, day 14 dbi was grouped with 0 dbi; in the second cluster, the 7 dai were grouped with the 21 dai. The different samples observed a differential pattern in global protein abundance levels. In this grouping, it can be seen that there is a greater tendency for poorly accumulated proteins in the first days. In contrast, highly accumulated proteins are more significant in the days after induction.

**Figure 3:**
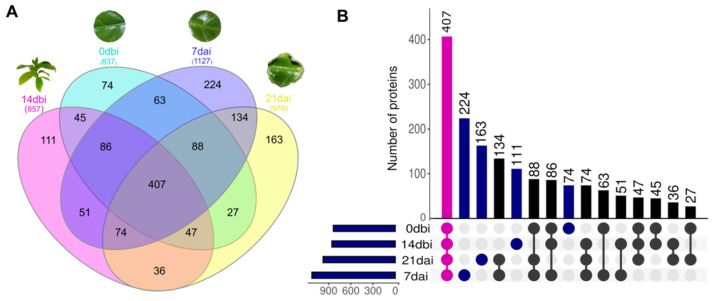
Comparison of total proteins among the different sampling days of somatic embryogenesis induction in *C. canephora*. (**A**) Venn diagram showing the distribution of 1630 proteins shared among the different days. (**B**) UpsetR plot. The overlapping regions correspond to the number of shared proteins between conditions. Magenta dots represent proteins identified in common across all days. Blue dots represent unique proteins for each day. dbi: days before induction. dai: days after induction.

Among the 1630 proteins identified, the highest number of accumulated proteins was found in the 7 dai samples, followed by 21 dai, 14 dbi, and 0 dbi (Figure 3). In the Venn diagram (Figure 3A) and the UpsetR plot (Figure 3B), the comparison of proteins between the different sampling days can be seen, as well as those proteins that are unique to each condition. On day 7 dai, there was a greater number of proteins exclusive to this day (224 proteins), while the smallest number of unique proteins was concentrated on day 0 dbi (74 proteins). On the other hand, 407 proteins were found accumulated on all sampling days, which can be considered constituent proteins of the SE process.

To perform the studies of differential accumulation analysis, the abundances of all proteins from days 0 dbi, 7 dai, and 21 dai were compared against those from 14 dbi. In this way, a total of three compared conditions were obtained: 0 dbi/14 dbi, 7 dai/14 dbi, and 21 dai/14 dbi.

**Figure 4:**
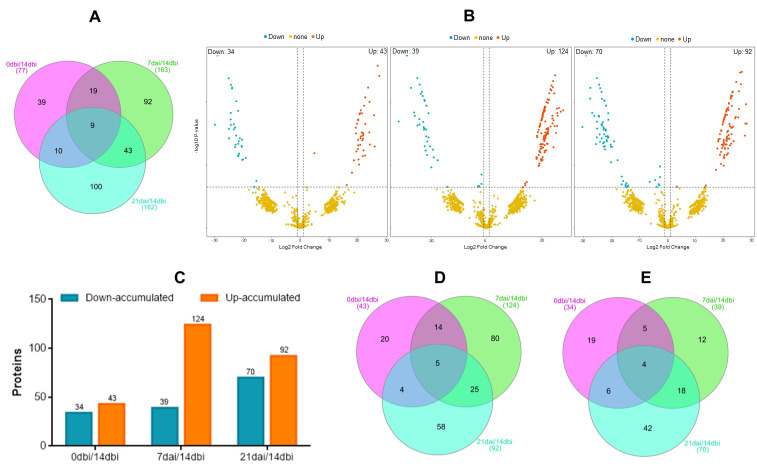
Differentially accumulated proteins (DAPs). (**A**) Venn diagram showing the total number of DAPs among the different days compared with 14 dbi (control): 0 dbi/14 dbi, 7 dai/14 dbi, and 21 dai/14 dbi. The overlapping regions correspond to the number of shared DAPs. (**B**) Volcano plots showing the distribution of DAPs among the different sampling days comparisons: 0 dbi/14 dbi, 7 dai/14 dbi, and 21 dai/14 dbi (panels from left to right). Down-accumulated proteins are indicated with blue dots on the left side of the plots. Up-accumulated proteins are indicated with orange dots on the right side of the plots. (**C**) Number of DAPs in the different comparisons. (**D**) Venn diagram of the up-accumulated DAPs (LFC ≥ 1; *p* ≤ 0.05). (**E**) Venn diagram of the down-accumulated DAPs (LFC ≤ 1; *p* ≤ 0.05).

The lowest number of differentially accumulated proteins (DAPs) was found in the 0 dbi/14 dbi condition, with 77 proteins (Figure 4A), followed by the 21 dai/14 dbi and 7 dai/14 dbi conditions, with 162 and 163 differential proteins, respectively. Of these proteins, 39 accumulated only in the 0 dbi/14 dbi condition, 92 in the 7 dai/14 dbi condition, and 100 in the 21 dai/14 dbi condition (Figure 4A).

Those differential proteins (*p* = 0.05) with a log fold change (LFC) greater than one were selected as up-accumulated, and those with an LFC greater than one were selected as down-accumulated (Figure 4B,C). In the 0 dbi/14 dbi condition, 43 up-accumulated and 34 down-accumulated proteins were found, with 20 (up-) and 19 (down-) proteins unique to this condition, respectively (Figure 4D,E). In the 7 dai/14 dbi condition, 124 up-accumulated and 39 down-accumulated proteins were found, of which 80 (up-) and 12 (down-) were unique to this comparison. On the other hand, in the 21 dai/14 dbi condition, 92 and 70 up and down-accumulated proteins were found, of which 58 (up-) and 42 (down-) were unique to this condition. The up-accumulated proteins from each comparison were selected for gene ontology analysis and KEGG pathway enrichment.

**Figure 5:**
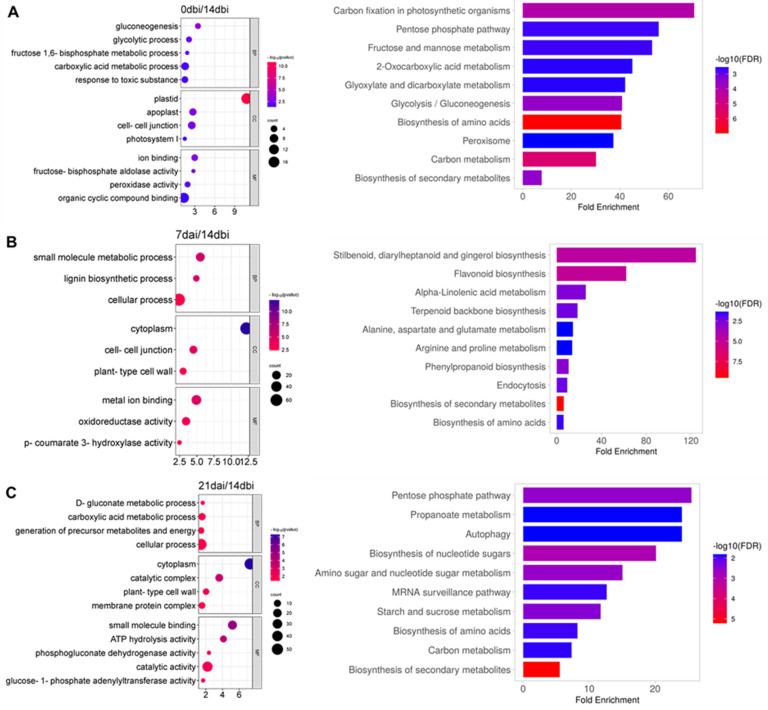
Gene enrichment analysis of up-accumulated DAPs. Gene ontology and KEGG enrichment of the most significant terms and pathways during (**A**) 0 dbi /14 dbi, (**B**) 7 dai/14 dbi, and (**C**) 21 dai/14 dbi comparison.

When comparing 0 dbi versus 14 dbi (Figure 5A), the most enriched biological processes were gluconeogenesis, glycolytic process, fructose 1,6-bisphosphate metabolic process, carboxylic acid process, and the response to toxic substances. The most significant cellular components were the plastid, apoplast, cell–cell junction, and photosystem I. The most enriched molecular functions were ion binding, fructose−bisphosphate aldolase activity, peroxidase activity, and organic cyclic compound binding. As for the KEGG pathways; some of the most significant routes were those related to carbon metabolism and biosynthesis of amino acids and secondary metabolites.

In the 7 dai/14 dbi comparison (Figure 5B), the most enriched terms corresponding to biological processes were the small molecule metabolic process, lignin biosynthetic process, and cellular process. The most significant cellular components were the cytoplasm, cell–cell junction, and plant cell wall. The most enriched molecular functions were metal ion binding, oxidoreductase activity, and coumarate hydroxylase activity. Among the different enriched KEGG pathways, we can highlight different amino acids biosynthesis, metabolism, and biosynthesis of secondary metabolites.

In the 21 dai/14 dbi condition (Figure 5C), the most important biological processes were gluconate and carboxylic acid metabolic processes, precursor metabolites, and energy generation. The most significant cellular components were the cytoplasm, catalytic complex, plant cell wall, and membrane protein complex. The small molecule binding, ATP hydrolysis, phosphogluconate dehydrogenase, and catalytic activity were the most enriched molecular functions. Once again, it was found that the biosynthesis of amino acids and secondary metabolites were some of the most enriched KEGG pathways, including autophagy and starch, sucrose, and carbon metabolism.

Once the global panorama of the biological processes carried out at the different points of the SE induction process was described, we focused on performing a manual search for all those proteins closely related to SE and plant development. We were able to identify 49 proteins accumulated on the four different sampling days, of which 18 were related to auxin, 2 to cytokinin (CK), 10 to SE process, 10 were 14-3-3 proteins, and 9 were serine/threonine protein phosphatases 2A (PP2A).

**Figure 6:**
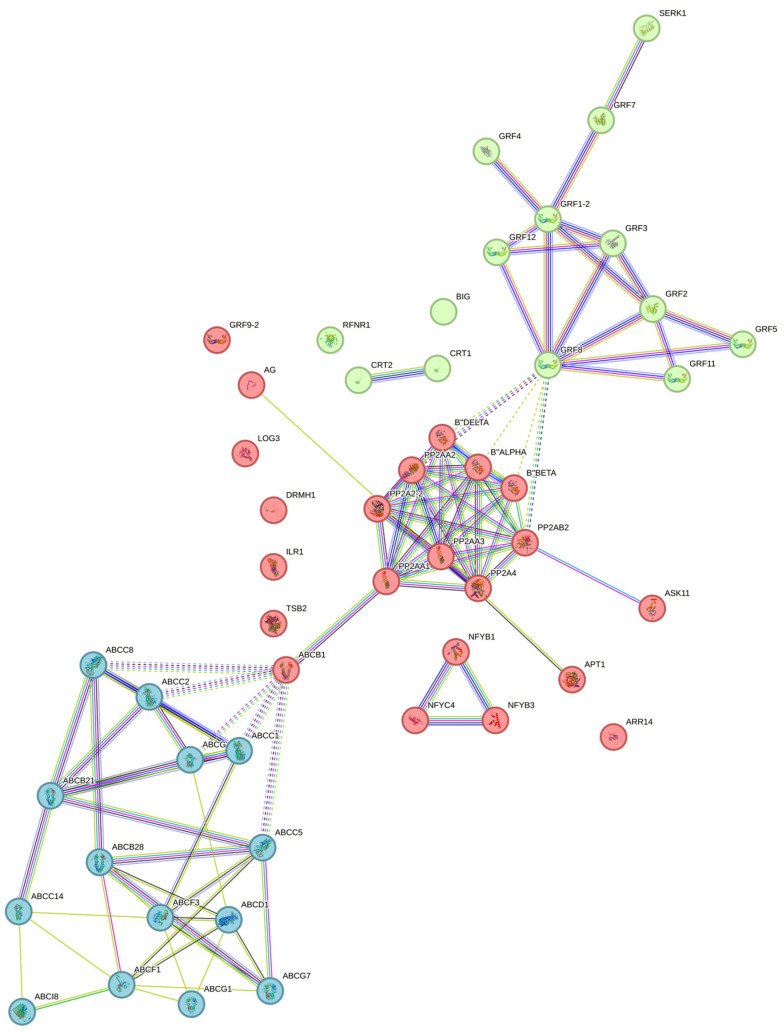
Interaction network of SE-related proteins in *C. canephora*. Kmeans clustering against *Arabidopsis thaliana* database. Edges represent protein–protein associations. The grouping of three large clusters are shown in different colors. Filled nodes mean that a 3D structure is known or predicted. The names and descriptions provided by STRING for each protein in the network are specified in Supplementary Table S1.

An interaction network was carried out (Figure 6) in which the grouping of three large clusters was observed: one composed mainly of the ATP-binding cassette (ABC) transporters, followed by another including the PP2A proteins, and the third cluster with the 14-3-3 proteins.

The ABC transporters of the different subfamilies interact with each other and are closely related. ABCB1 serves as a binding node with the cluster of PP2A proteins, which are connected to 14-3-3. Other essential proteins related to the metabolism of CK (CK) did not show a direct connection in this interactome.

**Figure 7:**
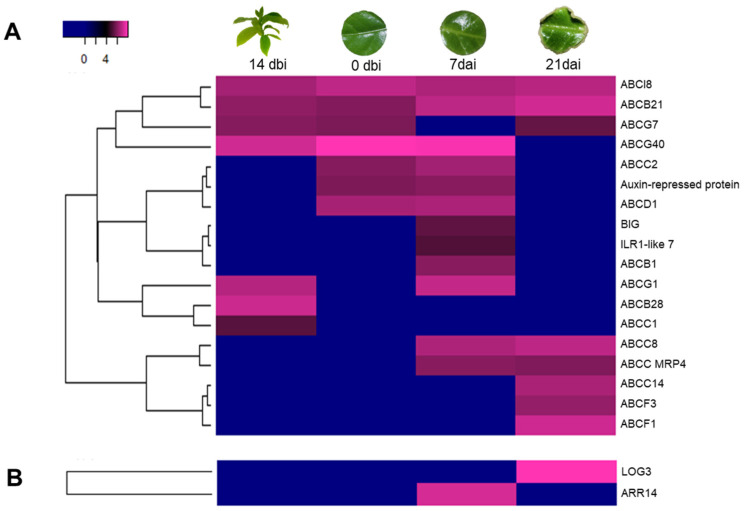
Accumulation profile of auxin- and cytokinin-related proteins during SE in *C. canephora*. (**A**) Auxin- and (**B**) Cytokinin-related proteins, among the different sampling days of the SE induction. Blue, black, and pink gradients represent low, intermediate, and high accumulation, respectively.

Proteins related to auxin homeostasis were selected (Figure 7A), which is crucial for the SE process. Within these, 15 proteins of the ABC transporter family were identified, belonging to the different subfamilies B, C, D, F, G, and I. Of these, ABCI8, ABCB21, and ABCG7 maintained a constantly high accumulation every sampling day. ABCB28 and ABCC1 were only accumulated on the control day (14 dbi). ABCB1 was only accumulated on day 7 dai.

On the other hand, ABCC14, ABCF3, and ABCF1 were the only ones highly accumulated on day 21 dai. Another transporter known as BIG protein was also observed, the only accumulation of which occurred on day 7 dai, as well as the ILR1-like 7 protein. This last one involved the hydrolysis of auxin conjugates with amino acids.

We could also identify two proteins closely related to the metabolism of CK (Figure 7B): the cytokinin-riboside 5′-monophosphate phosphoribohydrolase (LOG3) and the two-component response regulator (ARR14). LOG3 accumulated only on day 21 dai, while ARR14 did so on day 7 dai, both after induction with BA.

**Figure 8:**
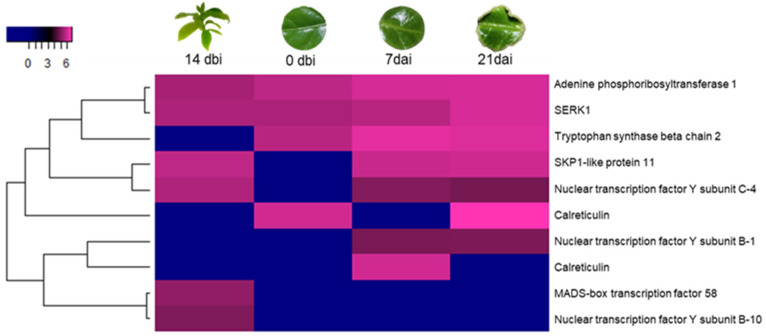
Accumulation profile of other SE-related proteins during SE in *C. canephora*. Blue, black, and pink gradients represent low, intermediate, and high accumulation, respectively.

Other SE-related proteins (Figure 8), such as the adenine phosphoribosyl transferase 1 and SE receptor kinase 1 (SERK1), were found to be accumulated throughout the entire process. The tryptophan synthase β-chain 2 showed no accumulation at 14 dbi. The SKP1-like protein 11 and the nuclear transcription factor Y subunit C-4 shared a similar pattern, with no accumulation at day 0 dbi. Calreticulins were observed at 0 dbi, 7 dai, and 21 dai. The nuclear transcription factor Y subunit B-1 was found only days after the induction. The MADS-box transcription factor 58 and the nuclear transcription factor Y subunit B-10 shared a similar pattern, being accumulated only on day 14 dbi.

**Figure 9:**
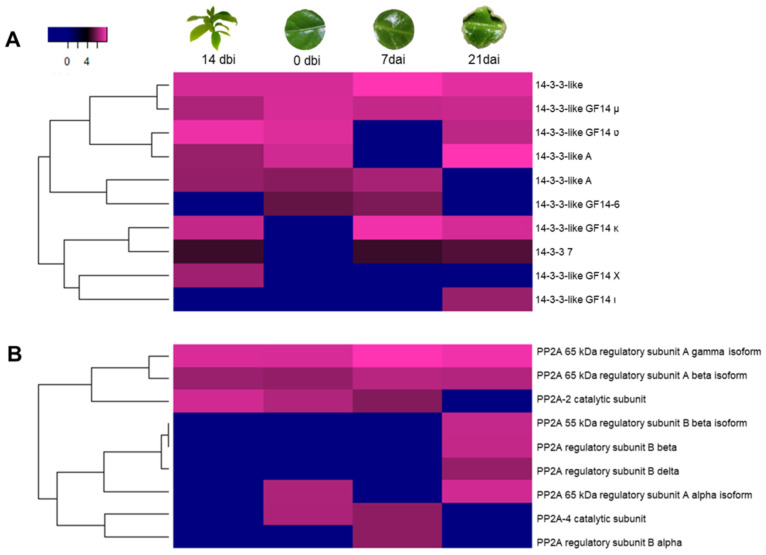
Accumulation profile of other proteins involved in plant development. (**A**) 14-3-3 and (**B**) PP2A proteins among the different sampling days of the SE induction. Blue, black, and pink gradients represent low, intermediate, and high accumulation, respectively.

Some 14-3-3 proteins are present during the induction of SE in different plant species, as was in our study model (Figure 9A). These proteins are strongly related to PP2A proteins (Figure 9B), which in turn are also linked to different processes of plant development, which will be discussed later. Two of the ten proteins in the 14-3-3 family found in our work (Figure 9A) were constantly accumulated throughout the process. The 14-3-3-like GF14 ʋ and 14-3-3-like-A shared a similar pattern, with no accumulation at 7 dai. 14-3-3-like GF14 κ and 14-3-3 7 showed no accumulation at 0 dbi. However, 14-3-3-like GF14 Χ protein was only accumulated at 14 dbi, while 14-3-3-like GF14 ι did so on the last day (21 dai).

Of the 9 PP2As (Figure 9B), those of the 65 kDa A γ and a β isoforms were highly abundant during the entire process. Both of the PP2A-2 catalytic subunits showed no accumulation at 21 dai. Those with the regulatory subunits B, β, and δ were only present at 21 dai, while the regulatory subunit B α was only found at 7 dai.

**Figure 10:**
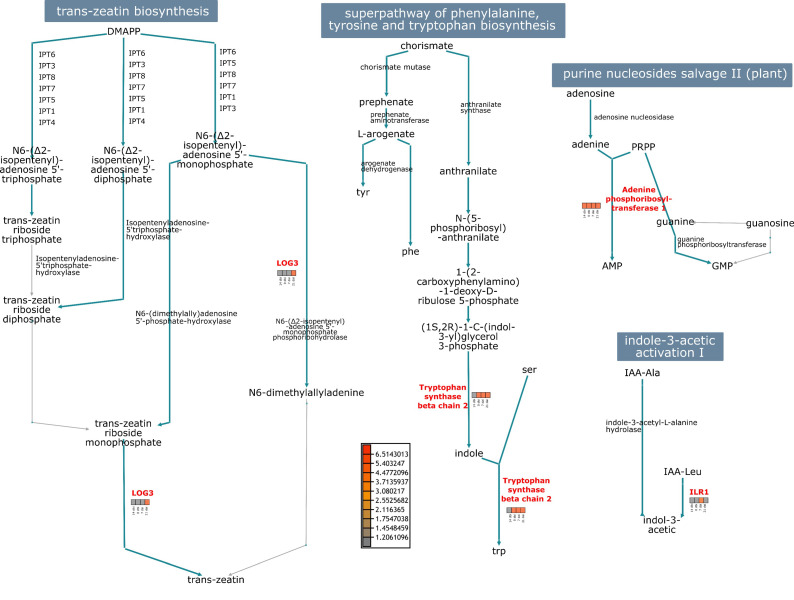
Participation of some SE-related proteins in different metabolic pathways. Abundances of LOG3, adenine phosphoribosyltransferase 1, ILR1, and tryptophan synthase proteins.

The abundance of proteins that seem to be related to SE was used to visualize the most significant metabolic pathways in which they intervene (Figure 10). The LOG3 protein intervenes in the trans-zeatin biosynthesis pathway during the conversion of *N*^6^-isopentenyl-adenosine-5′-monophosphate to *N*^6^-dimethylallyadenine; and from *trans*-zeatin riboside monophosphate to the final product: *trans*-zeatin. LOG3 was highly accumulated at the end of induction, where the first well-differentiated embryogenic structures were seen.

The adenine phosphoribosyltransferase 1 plays a significant role in the purine nucleosides salvage pathway. This protein catalyzes a salvage reaction involving adenine, resulting in the formation of AMP, and was highly accumulated throughout the entire process. In the indole-3-acetic acid pathway, ILR1 converted IAA-Leu conjugate to free IAA, its active form, and was highly accumulated in 7 dai. In the tryptophan biosynthesis pathway, we found the tryptophan synthase β chain 2, mediating the reaction from indole-3-glycerol phosphate to indole and tryptophan. This protein was accumulated on all days except for 14 dbi.

## 3. Discussion

*C. canephora* is a crop of great economic and cultural importance worldwide. SE has been a powerful biotechnology tool used in the *Coffea* genus, useful for carrying out large-scale genetic improvement and micropropagation studies to increase material in the field. However, although SE is a tissue culture tool widely used and studied for several decades, there are still many questions regarding its regulation, which would greatly help to manipulate and optimize the process and even to understand zygotic embryogenesis. New technologies allow the carrying out of studies at the genomic, transcriptomic, proteomic, and metabolomic levels and thus deepen research topics aimed at improving agriculture, the environment, human health, and biotechnology, among others [24,25,26]. Nowadays, many studies use transcriptomics to answer various biological questions. It allows for identifying the changes in the expression level of genes of interest in each condition and, therefore, understanding how the changes in the abundance of the transcripts control the growth and development of an organism [25,27].

Due to the global importance of the genus *Coffea*, there is a growing interest in the study and generation of transcriptomic data, specifically related to changes in the genetic program that allow a somatic cell to develop into an embryo [28]. A wide repertoire of work is aimed at unraveling somatic embryogenesis in *C. canephora*, addressing different strategies. However, the use of proteomic tools, such as shotgun and mass spectrometry, in this model is still scarce.

The closest proteomic study of SE induction in *C. canephora* was carried out by Mukul [23]. They found proteins that were secreted exclusively in the embryogenic condition and other proteins in the non-embryogenic condition. However, the identity of these proteins was not established.

**Figure 11:**
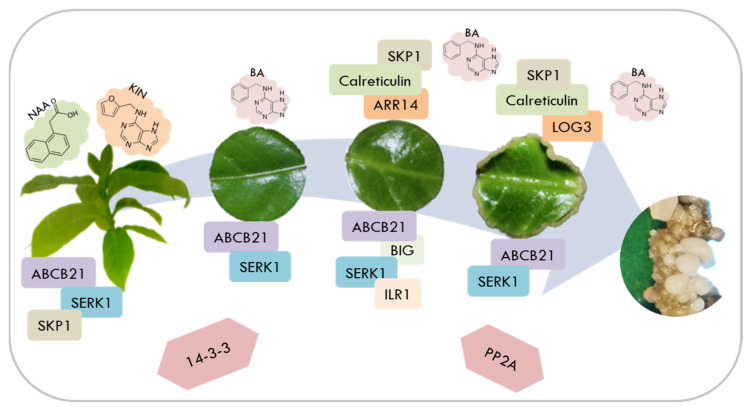
Model of SE induction in *C. canephora* and the main proteins found to be involved during the process.

During SE of *C. canephora*, it was previously shown that the exogenous addition of PGR is crucial for forming the first embryogenic structures. In this process, there are dynamic changes in the endogenous pools of auxin [29] and CK [30]. The content of auxin and its conjugates increases during pretreatment, while the expression of different genes involved in auxin homeostasis increases, such as YUCCA [29], GH3 [31], PIN [32], ARF, and Aux/IAA [13]. However, during the induction stage, IAA levels decrease while an increase in the expression of CK signaling genes is observed. There is a mobilization of auxin from the chloroplast to the growing areas PIN [32]. Now, by implementing proteomics in this same study model, we were able to identify and quantify some of the proteins that could be vigorously participating in the induction of SE. Thus, a model was proposed where the proteins most related to the SE induction process in *C. canephora* are summarized (Figure 11) and later discussed.

One of the crucial processes during SE is the transport of auxin. This transport is mediated by different families of proteins, among which the ABC transporters stand out. ABC transporters’ family is ubiquitous and divided into eight groups: A–G and I [33]. They regulate the transport of auxin and other molecules, such as lipids, sugars, and polysaccharides. This work identified ABCs belonging to families B, C, D, F, G, and I.

The ABCs in which the relationship with IAA has been demonstrated belong to subfamily B: ABCB1, ABCB4, ABCB19, and ABCB21 [34]. We found that ABCB21 was accumulated on each sampling day, while ABCB1 was only accumulated seven days after induction. These two proteins were crucial players in the SE of *Lilium pumilum* [35]. It is also essential in cell differentiation, as found in in vitro tissues of *C. arabica* [36]. The only ABC proteins detected on day 21 were ABCC14, ABCF3, and ABCF1. However, in plants, there are only records of the function of the first two. ABCC14 has been found in response to stressful conditions and possibly involved in transporting heavy metals such as tomatoes [37] and peanuts [38]. For its part, ABCF3 seems to be involved in the development of thylakoids in chloroplasts [39]. CK, like auxins, can be transported by large families of proteins, including PUP, ENT, and ABC subfamily G. In our model, we identified some of them, but there are no previous reports of their participation in other plant species.

There are few studies where the BIG protein has been identified in different processes of plant development [40], tissue differentiation [36], and the coordination of some PGR pathways [41]. Its function is associated with the polar transport of auxin, in addition to participating in vesicular trafficking and targeting of auxin transporters such as PINs in the endocytic pathway cycling, and as a mediator of auxin in pericycle cell activation promoting root hair elongation [40,42,43]. However, we have not found any study that demonstrates its effect on SE [44]. One way to regulate endogenous IAA levels is via conjugates with amino acids. When the cell requires free auxin, the conjugates can be hydrolyzed by amidohydrolases such as IAA-LEUCINE RESISTANT1 (ILR1) or ILR1-LIKE (ILL) to return to their active form. In cotton, a decrease in ILR/ILL expression was observed as the SE induction process progressed and until the development of the embryos, as occurred in our model [45]. It is also worth mentioning that during the induction stage, proteins related to auxin signaling, such as SKP1 and the 26S proteasome, were found. The SKP1 protein is part of the SCF complex where the auxin is perceived, and the Aux/IAA proteins are ubiquitinated and degraded via the 26S proteasome. This way, the transcription of auxin-responsive genes that participate in SE occurs. All of the above could suggest that the ABC, BIG, and ILR1 proteins found on 7 dai are important in regulating auxin flow, leading to the future formation of embryogenic structures at 21 dai.

In the case of auxins, endogenous IAA increases in response to adding exogenous PGR to the culture medium. However, in CK, this panorama has not been fully demonstrated [30,46]. Identifying the accumulation of proteins related to CK metabolism during the induction stage, in which BA is added to the culture medium to initiate SE, could suggest that endogenous changes in these PGRs also occur in response to their exogenous addition.

The genes LONELY GUY (LOG) and ARABIDOPSIS RESPONSE REGULATOR (ARR) are within the components of CK homeostasis. The first ones are involved in nucleotide activation. That is, in the same way that occurs in auxins, the active form of CK can be obtained via the synthesis of cytokinin−ribotides mediated by the cytokinin−riboside 5′-monophosphate, also known as LOG [47]. In *A. thaliana*, LOG3 expression levels gradually increased towards the last day of induction, as occurred in our model at day 21 dai [46]. On the other hand, ARRs participate in CK signaling. In previous work in our laboratory [30], various member genes of the ARR family were detected, the majority of which had a high expression on the first days after induction, while on the last day (21 days), this expression was considerably reduced. Again, we detected the same pattern in this study, but now with a proteomic approach.

Although a differential accumulation of ADENINE PHOSPHORIBOSYL TRANSFERASE (APRT) was not observed, it is important to mention its role in other plant development processes and SE. This enzyme converts adenine to AMP in a single step and is part of plants’ purine nucleosides salvage pathway. APRTs recycle adenine into adenylate nucleotides. They can also use CK as substrates since they are adenine derivatives with N^6^ substitutions (side chains of different lengths and structures), which control their biological action. It has been hypothesized that CK biosynthesis and their interconversion depend on APT activity [48]. As in the case of auxins, CK also seems to regulate their active form [49]. In *Picea glauca*, fluctuations in the components of the purine salvage pathway were found [50]. The above suggests a key point of regulation that determines the end of cell proliferation of the proembryogenic tissue and the beginning of embryo development.

We found a highly accumulated calreticulin on day 21 dai. Calreticulin was discovered by analyzing Ca^2+^-associated proteins in spinach, and a high homology was subsequently found between its counterparts in mammals. The biological function of these proteins is inferred to be the regulation of Ca^2+^ signaling, modulation of gene expression, and molecular chaperones [51]. There are no current reports that report its participation in the SE. However, studies carried out on *Nicotiana plumbaginifolia* [52] and *Dacus carota* [53] a few decades ago reported an essential activity of calreticulin in zygotic (ZE) and SE. In *Nicotiana*, maximum calreticulin activity was obtained during the early stages of the SE and in response to auxin, while in the ZE it accumulated in the embryo proper. In *Dacus*, the accumulation of this and other Ca^2+^-associated proteins was localized in the protoderm of somatic embryos. With the above, it was concluded that the function of calmodulin in embryogenesis is to bind Ca^2+^ and store it for the correct development of the embryos.

One of the main transcription factors (TF) determining SE is somatic embryogenesis receptor kinase 1 (SERK1) [4]. In *Araucaria angustifolia*, this TF was expressed in the periphery of the embryogenic callus and later in the embryo proper [54]. Previous studies carried out in *C. canephora* [55] reported that overexpression of SERK1 caused an increase in the number of somatic embryos, concluding that this TF regulates the induction of SE via the activation of auxin homeostasis genes. Additionally, 14-3-3 proteins interact with SERK1 to enhance embryogenic competence [56]. Using quantitative proteomics, it is possible to determine that 14-3-3 proteins, via the regulation of ATP synthases, participate in the first stages of SE in response to exogenous PGR [57]. PP2A proteins regulate histone modifications and gene expression, which are essential for forming and developing somatic embryos [4].

We also identified a nuclear transcription factor Y subunit B-C on induction days. However, no reports of this particular TF and its relationship with SE exist. On the contrary, it has been shown that nuclear transcription factor Y subunit alpha (NFYA) participates in embryogenesis. This family of TFs is not well characterized in plants, but a few studies suggest that it is a stress- and PGR-responsive TF closely linked to ES and embryo development [58].

Proteomics, in conjunction with other omics and molecular tools, can provide novel information for understanding the functioning of the SE process.

## 4. Materials and Methods

### 4.1. Biological Material and Growth Conditions

Plantlets of *C. canephora* grown in vitro were used as initial biological material. These plantlets were subcultured every six weeks in a semi-solid maintenance medium without PGR [MS salts (PhytoTechnology Laboratories, M524), 11.85 µM thiamine-HCl (Sigma, T3902), 550 µM myo-inositol (Sigma, I5125), 158 µM cysteine hydrochloride hydrate (Sigma, C121800), 16.24 µM nicotinic acid (Sigma, N4126), 9.72 µM pyridoxine-HCl (Sigma, P9755), 87.64 mM sucrose (Sigma, S539) and 0.285% (*w*/*v*) Gellan gum (PhytoTechnology Laboratories, G434), adjusted to pH 5.8]. Plantlets were incubated under a photoperiod of 16 h light and 8 h dark at 25 ± 2 °C.

For the SE induction process, the seedlings were previously incubated for 14 d in a preconditioning semi-solid medium [same composition of the maintenance medium, added with 0.54 µM naphthaleneacetic acid (NAA; Sigma, N1145; St. Louis, MO, USA) and 2.32 µM kinetin (KIN; Sigma, K0753; St. Louis, MO, USA) adjusted to pH 5.8]. After 14 d of preconditioning, we cut circular explants from the second and third pair of leaves in a basipetal direction with a sterile punch of 0.8 mm in diameter.

Five explants were placed in 50 mL induction liquid culture medium [Yasuda salts [59] supplemented with 5 µM 6-benzyladenine (BA; PhytoTechnology Laboratories, B800; Lenexa, KS, USA) and adjusted to pH 5.8] in 250 mL flasks. The explants were incubated in the dark at 25 ± 2 °C and shaking (60 rpm). Samples were taken for subsequent analysis during preconditioning (14 and 0 dbi) and after induction of SE (7 and 21 dai). For sampling, sections of leaf discs were used in all samples, including the control day, so that the comparison between them was homogeneous. The leaf discs were briefly rinsed with distilled water to remove excess culture medium and placed on absorbent paper to remove liquid on the explant surfaces. They were subsequently weighed into 100 mg packets per triplicate, frozen in liquid nitrogen, and then stored at −80 °C until use.

### 4.2. Protein Extraction

An amount of 100 mg of plantlet leaves was pulverized in a mortar with liquid nitrogen until a fine powder, avoiding thawing. The samples were always kept cool during extraction. The sample powder was transferred to a 2 mL microcentrifuge tube, and 1 mL of extraction buffer was added, containing 0.5 M Trizma base (pH 8; Sigma, T1503; St. Louis, MO, USA), 50 mM EDTA (Sigma, EDS; St. Louis, MO, USA), 0.7 M sucrose, 0.1 M KCl (Sigma, P9541; St. Louis, MO, USA), 50 mM DTT (Sigma, D5545; St. Louis, MO, USA), 1% SDS (Sigma, L3771; St. Louis, MO, USA), 1 mM PMSF (Sigma, 78830; St. Louis, MO, USA) and a protease inhibitor cocktail (Sigma, P9599; St. Louis, MO, USA). The extract was vigorously vortexed for 2 min, followed by 15 min ice incubation with gentle shaking. An equivalent volume of phenol solution (Sigma, P4557; St. Louis, MO, USA) was added and vigorously vortexed, followed by incubation on ice with gentle shaking for 30 min. The tubes were centrifuged at 15,000× *g* for 30 min at 4 °C. The upper phase was recovered in a new tube, avoiding carrying cell debris. Proteins were precipitated overnight at −20 °C with five volumes of 0.1 M ammonium acetate (CTR, 00140; Monterrey, NL, México) dissolved in methanol with 5 mM DTT. The following day, tubes were centrifuged at 15,000× *g* for 30 min at 4 °C, and the supernatant was discarded. The protein pellet was washed once with 1 mL 0.1 M ammonium acetate/methanol/5 mM DTT and twice with 80% acetone/5 mM DTT. The pellet was allowed to dry in an extraction hood for 3–5 min and then resuspended in 50 mM ammonium bicarbonate (Sigma, A6141; St. Louis, MO, USA) supplemented with 0.1% SDS (Sigma, L3771; St. Louis, MO, USA) until completely dissolved. Protein quantitation was determined by the Peterson method [60], and the quality was visualized on SDS-PAGE.

### 4.3. Reduction, Alkylation, and Digestion

For sample reduction, 10 mM TCEP (Sigma, 68957; St. Louis, MO, USA) was added and incubated at 60 °C for 45 min. For alkylation, 30 mM IAM (Sigma, A3221; St. Louis, MO, USA) was added and incubated for 60 min in the dark at room temperature. Then, 30 mM DTT was added and incubated for 10 min. Proteins were precipitated with cold acetone overnight at −20 °C. The next day, the tubes were centrifuged at 10,000× *g* for 15 min at 4 °C, and the supernatant was discarded. The pellet was allowed to dry in an extraction hood for 3–5 min and resuspended in 50 mM ammonium bicarbonate with 0.1% SDS. Protein concentration was quantified, and quality was visualized on SDS-PAGE. Digestion was carried out with trypsin (Thermo Scientific, 90058; Rockford, IL, USA) in 150 µg of protein at a 1:60 ratio (trypsin/protein) overnight at 37 °C without rotation. The next day, more trypsin was added at 1:100 for 4 h.

### 4.4. Nano LC/MS-MS Analysis

The preparation of two biological replicates per sample and subsequent analysis was carried out, as reported by Bautista [61]. The fractionation was carried out offline before LC-MS/MS analysis with high pH reversed-phase liquid chromatography spin columns (Pierce High pH Reversed-Phase Cat No. 84868). Three fractions were obtained after elution with increasing concentrations of acetonitrile (15, 17, and 20%). Then, fractions were desalted using ZipTip-C_18_ tips (Merck Millipore, Darmstadt, Germany) and dried in a vacuum concentrator. An Orbitrap Fusion Tribrid (Thermo-Fisher Scientific, San Jose, CA, USA) mass spectrometer equipped with an “EASY spray” nano ion source (Thermo-Fisher Scientific, San Jose, CA, USA) and interfaced with an UltiMate 3000 RSLC system (Dionex, Sunnyvale, CA, USA) was used to analyze the samples. Each sample was reconstituted with 0.1% formic acid in LC-MS grade water (solvent A). Five μL were injected into a nanoviper C_18_ trap column (3 μm, 75 μm × 2 cm, Dionex) at a flow rate of 3 μL min^−1^ and separated on an EASY spray C-_18_ RSLC column (2 μm, 75 μm × 25 cm). A 100 min gradient of Solvent A and 0.1% formic acid in 90% acetonitrile (Solvent B) with a 300 nL min^−1^ flow rate was used as follows: 10 min with 100% solvent A, 25 min with 7–20% solvent B, 15 min with 20% solvent B, 15 min with 20–25% solvent B, 20 min with 25–95% solvent B, 8 min with solvent A. The mass spectrometer was set to positive ion mode, with a nanospray voltage of 3.5 kV and a source temperature of 280 °C; precursor selection mass range of 400–1200 *m*/*z*, precursor ion exclusion width of low 18 *m*/*z* and high 5 *m*/*z*, The external calibrants were Caffeine, Met-Arg-Phe-Ala (MRFA), and Ultramark 1621 (Cat No. 88323, Thermo-Fisher Scientific Pierce). MIAPE Reporting guidelines for mass spectrometry are described in Supplementary Materials S1. The mass spectrometry proteomics data were deposited to the ProteomeXchange Consortium via the PRIDE [62] partner repository (http://www.ebi.ac.uk/pride; accessed on 20 November 2023) with the dataset identifier PXD047172.

### 4.5. Data Processing

The resulting MS/MS data were processed using the MASCOT (v.2.4.1, Matrix, Science, Boston, MA, USA) search engine implemented in Proteome Discoverer 2.2 (PD, Thermo Fisher Scientific, San Jose, CA, USA). The PD parameters were set as follows: *viridiplantae* Swiss-prot database, mass tolerance of 10 ppm and 0.6 Da, two missed cleavages allowed, 0.01 FDR, cysteine carbamidomethylation as fixed modification, methionine oxidation and N-terminal acetylation as dynamic modification. For label-free quantification, the Minora node was incorporated into the Processing workflow. The output data were filtered, eliminating those rows with empty abundance values. The filtered results in the spreadsheets were uploaded to the Galaxy platform for the differential analysis, and the Limma tool and normalization by TMM were used. Differentially abundant proteins were selected according to a log fold change of 1 and *p* < 0.05. Annotation was carried out in KOBAS against the *Arabidopsis* database. Protein IDs were loaded on g:Profiler (https://biit.cs.ut.ee/gprofiler/gost; accessed on 21 August 2023) to perform GO enrichment. ShinyGO v0.77 (http://bioinformatics.sdstate.edu/go/; accessed on 21 August 2023) was used to perform KEGG enrichment. The protein–protein interaction network of SE-related proteins was carried out using STRING (https://string-db.org/; accessed in 21 August 2023). PlantCyc (https://pmn.plantcyc.org/; accessed on 1 September 2023) was used to visualize SE-related protein abundances in the different biochemical pathways.

## 5. Conclusions

The addition of growth regulators exogenously favors the induction of somatic embryos via a series of response reactions to this stimulus. On day 7, the greatest number of accumulated proteins was observed; this is possibly due to the fact that various metabolic and cellular changes are occurring in the explant to give way to the formation of embryogenic structures that will be visible on day 21. In our model, proteins involved in the metabolism of auxin and CK were observed throughout the process. The above is an indication of crosstalk between both regulators. Proteins of the ABC and BIG family indicate active mobilization of IAA, while ILR1 would participate in the hydrolysis of IAA conjugates. On the other hand, the ARR and LOG proteins demonstrate that there are active CK signaling and activation pathways. Other proteins involved in SE and diverse processes of plant development were also confirmed, such as 14-3-3, PP2A, SKP1, and calreticulin, as well as some transcription factors like SERK1. There is no previous record of the proteomic study of SE induction in *C. canephora*. Hence, our results provide basic information to better understand the SE mechanism in *C. canephora* using proteomic tools and lay the foundations for future more in-depth work. Proteomics, in conjunction with other omics and molecular tools, can provide novel information for understanding the functioning of the SE process.

## Data Availability

Data are available via ProteomeXchange with the identifier PXD047172.

## Supplementary Materials

The following supporting information can be downloaded at https://www.mdpi.com/article/10.3390/plants12244095/s1. Table S1: Description of protein–protein interaction network of SE-related proteins. Supplementary Material S1: Reporting guidelines for mass spectrometry.
