# Supplementary material for: Proteomic Approach during the Induction of Somatic Embryogenesis in Coffea canephora
Source: Plants (Basel). 2023 Dec 7;12(24):4095. doi: 10.3390/plants12244095 (PMC10748034; doi:10.3390/plants12244095)
Supplement: Supplementary file 1 [file plants-12-04095-s001.zip › Supplementary 1.pdf]

Reporting guidelines for mass spectrometry

1. General Features

1.1 Global descriptors

- Responsible person (or institutional role if more appropriate); provide name, affiliation, and stable contact information: Eliel Ruiz May/José Miguel Elizalde Contreras. Red de Estudios Moleculares Avanzados, Clúster Científico y Tecnológico BioMimic®, Instituto de Ecología A.C. (INECOL), Carretera Antigua a Coatepec No. 351, Congregación el Haya, CP 91070, Xalapa, Veracruz, México. eliel.ruiz@inecol.mx.
- Instrument manufacturer and model: Orbitrap Fusion Tribrid (Thermo-Fisher Scientific, San Jose, CA, USA) mass spectrometer.
- Customizations (summary): Global Settings

Method Duration (min)= 120

Ion Source Type = NSI

Spray Voltage: Positive Ion (V) = 3500

Spray Voltage: Negative Ion (V) = 600

Sweep Gas (Arb) = 0

Ion Transfer Tube Temp (°C) = 280

Internal Mass Calibration= Easy-IC

Pressure Mode = Standard

Default Charge State = 2

Experiment 1

Start Time (min) = 0

End Time (min) = 120

Cycle Time (sec) = 3

Do data dependent experiment if no target species are found = False

Scan MasterScan MSn Level = 1

Use Wide Quad Isolation = True

Detector Type = Orbitrap

Orbitrap Resolution = 120K

Mass Range = Normal

Scan Range (m/z) = 350-1500

Maximum Injection Time (ms) = 50

AGC Target = 400000

Microscans = 1

S-Lens RF Level = 60

Use ETD Internal Calibration = True

DataType = Profile

Polarity = Positive
Source Fragmentation = False
Filter MIPS
Filter Type = MIPS
MIPS On = 2
Relax Restrictions = True
Filter ChargeState
Filter Type = ChargeState
Include charge state(s) = 2-8
Include undetermined charge states = False
Include charge states 25 and higher = False
Filter DynamicExclusion
Filter Type = DynamicExclusion
Exclude after n times = 1
Exclude isotopes = True
Perform dependent scan on single charge state per precursor only = False
If occurs within (s) = 30
Exclusion duration (s) = 90
Excl. Mass Width = ppm
Mass tolerance low = 10
Mass tolerance high = 10
Filter IntensityThreshold
Filter Type = IntensityThreshold
Signal Intensity = 10000
Decision
Precursor Priority = MostIntense
Scan Event 1
ChargeRange: 3-3
AND
MZRange: 300-1600
OR
ChargeRange: 4-4
AND
MZRange: 300-1600
OR
ChargeRange: 5-5
AND
MZRange: 300-1600

OR

ChargeRange: 6-8

Scan Event 2

ChargeRange: 2-2

OR

ChargeRange: 3-3

AND

MZRange: 300-1600

OR

ChargeRange: 4-4

AND

MZRange: 300-1600

OR

ChargeRange: 5-5

AND

MZRange: 300-1600

OR

ChargeRange: 6-8

Scan Event 1

Do data dependent experiment if no target species are found = False

Scan ddMSnScan MSn Level = 2

Top N= 0

Isolation Mode = Quadrupole

Isolation Window = 1.6

Scan Range Mode = Auto Normal FirstMass = 120

ActivationType = HCD

Is Stepped Collision Energy On = False

Stepped Collision Energy (%) = 5

Multistage Activation = False

Neutral Loss Mass = 50.0001

Collision Energy (%) = 28

Detector Type = Orbitrap

Orbitrap Resolution = 30K

Maximum Injection Time (ms) = 50

AGC Target = 50000

Inject ions for all available parallelizable time = True Microscans = 1

Activation Q = 0.25

Use ETD Internal Calibration = False

DataType = Centroid
Polarity = Positive
Source Fragmentation = False
Scan Event 2
Do data dependent experiment if no target species are found = False
Scan ddMSnScan MSn Level = 2
Isolation Mode = Quadrupole
Top N= 0
Isolation Window = 1.6
Use Isolation m/z Offset = False
Multi-notch Isolation = False
Scan Range Mode = Auto Normal FirstMass = 100
ActivationType = CID Collision Energy (%) = 35
Neutral Loss Mass = 50.0001
Is Stepped Collision Energy On = False
Stepped Collision Energy (%) = 5
Multistage Activation = False
Is EThcD Active = False
Detector Type = Orbitrap
Orbitrap Resolution = 30K
Maximum Injection Time (ms) = 50
AGC Target = 50000
Inject ions for all available parallelizable time = True Microscans = 1
Activation Q = 0.25
DataType = Centroid
Polarity = Positive
Source Fragmentation = False
HPLC
Run time: 121.000 [min]
Instrument: MININT-82L3M2J_1 on minint-82l3m2j Description:
initial Instrument Setup: PumpModule.LoadingPump.%A.Equate: "H2O +0.1% Formic Acid", PumpModule.LoadingPump.%B.Equate: "ACN +0.1%Formic Acid", PumpModule.LoadingPump.%C.Equate: "%C", PumpModule.NC_Pump.%A.Equate: "%A" H2O +0.1% Formic Acid, PumpModule.NC_Pump.%B.Equate: "%B" ACN +0.1% Formic Acid
-20.000 [min] Equilibration, PumpModule.LoadingPump.Flow.Nominal: 3.000 [µl/min] PumpModule.LoadingPump.%B.Value: 0.0 [%] PumpModule.LoadingPump.%C.Value: 0.0 [%] PumpModule.LoadingPump.Curve:5,

PumpModule.NC_Pump.Flow.Nominal: 0.250 [µl/min] PumpModule.NC_Pump.
%B.Value: 7.0 [%], PumpModule.NC_Pump.Curve: 5
0.000 [min] Inject Preparation, Wait PumpModule.LoadingPump.Ready And PumpModule.NC_Pump.Ready And ColumnOven.Ready And Sampler.Ready 0.000 [min] Inject, Sampler.Inject
0.000 [min] Start Run, ColumnOven.ColumnOven_Temp.AcqOn, PumpModule.LoadingPump.LoadingPump_Pressure.AcqOn, PumpModule.NC_Pump.NC_Pump_Flow.AcqOn,PumpModule.NC_Pump.NC_Pump_Flow_LeftBlk.AcqOn, PumpModule.NC_Pump.NC_Pump_Flow_RightBlk.AcqOn, PumpModule.NC_Pump.NC_Pump_Pressure.AcqOn
0.000 [min] Run PumpModule.LoadingPump.Flow.Nominal: 3.000 [µl/min], PumpModule.LoadingPump.%B.Value: 0.0 [%] PumpModule.LoadingPump.
%C.Value: 0.0 [%], PumpModule.LoadingPump.Curve: 5,
PumpModule.NC_Pump.Flow.Nominal: 0.250 [µl/min], PumpModule.NC_Pump.
%B.Value: 7.0 [%], PumpModule.NC_Pump.Curve: 5
10.000 [min] PumpModule.NC_Pump.Flow.Nominal: 0.250 [µl/min], PumpModule.NC_Pump.%B.Value: 7.0 [%], PumpModule.NC_Pump.Curve: 5, ColumnOven.ValveRight: 10_1
20.000 [min] PumpModule.LoadingPump.Flow.Nominal: 3.000 [µl/min], PumpModule.LoadingPump.%B.Value: 0.0 [%], PumpModule.LoadingPump.%C.Value: 0.0 [%], PumpModule.LoadingPump.Curve: 5
30.000 [min] PumpModule.LoadingPump.Flow.Nominal: 0.300 [µl/min], PumpModule.LoadingPump.%B.Value: 50.0 [%], PumpModule.LoadingPump.
%C.Value: 0.0 [%], PumpModule.LoadingPump.Curve: 5
35.000 [min] PumpModule.NC_Pump.Flow.Nominal: 0.250 [µl/min], PumpModule.NC_Pump.%B.Value: 19.0 [%], PumpModule.NC_Pump.Curve: 5
50.000 [min] PumpModule.NC_Pump.Flow.Nominal: 0.250 [µl/min], PumpModule.NC_Pump.%B.Value: 20.0 [%], PumpModule.NC_Pump.Curve: 5
65.000 [min] PumpModule.NC_Pump.Flow.Nominal: 0.250 [µl/min], PumpModule.NC_Pump.%B.Value: 25.0 [%], PumpModule.NC_Pump.Curve: 5
80.000 [min] PumpModule.LoadingPump.Flow.Nominal: 0.300 [µl/min], PumpModule.LoadingPump.%B.Value: 25.0 [%], PumpModule.LoadingPump.
%C.Value: 0.0 [%], PumpModule.LoadingPump.Curve: 5
86.000 [min] PumpModule.NC_Pump.Flow.Nominal: 0.250 [µl/min], PumpModule.NC_Pump.%B.Value: 95.0 [%], PumpModule.NC_Pump.Curve: 9
90.000 [min] PumpModule.LoadingPump.Flow.Nominal: 3.000 [µl/min], PumpModule.LoadingPump.%B.Value: 0.0 [%], PumpModule.LoadingPump.%C.Value: 0.0 [%], PumpModule.LoadingPump.Curve: 5

94.000 [min] PumpModule.NC_Pump.Flow.Nominal: 0.250 [µl/min], PumpModule. NC_Pump.%B.Value: 95.0 [%], PumpModule. NC_Pump.Curve: 5
100.000 [min] PumpModule.NC_ Pump.Flow.Nominal: 0.250 [µl/min], PumpModule.NC_Pump.%B.Value: 7.0 [%], PumpModule.NC_Pump.Curve: 5
120.000 [min] PumpModule.LoadingPump.Flow.Nominal: 3.000 [µl/min], PumpModule. LoadingPump.%B.Value: 0.0 [%], PumpModule.LoadingPump.%C.Value: 0.0 [%], PumpModule. LoadingPump.Curve: 5, PumpModule.NC_Pump.Flow.Nominal: 0.250 [µl/min], PumpModule.NC_Pump.%B.Value: 7.0 [%], PumpModule.NC_Pump.Curve: 5, ColumnOven.ValveRight: 1_2
121.000 [min] Stop Run

2. Ion sources

As each spectrum is acquired using only one ionization source, select the one that applies

2.1 Electrospray Ionization (ESI)

- Supply type (static or fed): Static.
- Interface manufacturer, model: EASY-Spray™ ES081
- Sprayer type, manufacturer, model: EASY spray" nano ion source (Thermo-Fisher Scientific, San Jose, CA, USA) and interfaced with an UltiMate 3000 RSLC system (Dionex, Sunnyvale, CA, USA).
- Other parameters if discriminant for the experiment: NA

2.2 MALDI

- Plate composition (or type): NA
- Matrix composition: NA
- PSD (or LID/ISD) summary, if performed: NA
- Laser type and wavelength: NA
- Other laser and source-related parameters, if discriminating for the experiment: NA

2.3 Other ionization source

- Description of the ion source and relevant parameters: NA

3. Post-source component

As an MS spectrum or chromatogram performed on one instrument cannot be acquired using all existing analysers and detectors, select the elements that apply.

3.1 Analysers

- Ion optics, 'simple' quadrupole, hexapole, Paul trap, linear trap, magnetic sector, FT- ICR, Orbitrap: name of the analysers(s): Orbitrap Fusion Tribrid
- Time-of-flight drift tube (TOF): Reflectron status: NA

3.2 Activation / dissociation

The associated acquisition parameters are covered in 4.1

- Instrument component where the activation/dissociation occurs: Ion-Routing Multipole/Dual Pressure Linear IonTrap
- Gas type (when used): Helium
- Activation/dissociation type: CID, HCD

4. Spectrum and peak list generation and annotation

4.1 Data acquisition

- Software name and version: Xcalibur 4.0.27.10

4.2 Data analysis

- Software name and version: Proteome Discoverer 2.2 (Thermo Fisher Scientific, San Jose, CA, USA)
- Parameters used in the generation of peak lists or processed spectra: mass tolerance of 10 ppm and 0.6 Da, two missed cleavages allowed, 0.01 FDR, cysteine carbamidomethylation as fixed modification, methionine oxidation and N-terminal acetylation as dynamic modification.

4.3 Resulting data

- Location of source (‚raw‘) and processed files: ProteomeXchange repository with ID PXD047172 and DOI 10.6019/PXD047172
- The chromatogram(s) for SRM data and other relevant cases: NA
- m/z and intensity values: 350–1500 m/z, intensity threshold of 5.0 e3.
- MS level: MS2
- Ion mode: positive ion mode
- For MS level 2 and higher, precursor m/z and charge, if known, with the full mass spectrum/peak list containing that precursor peak, where available: precursor selection mass range of 400–1200 m/z, precursor ion exclusion width of low 18 m/z and high 5 m/z, data will be available at ProteomeXchange repository with ID PXD047172 and DOI 10.6019/PXD047172
