# Supplementary material for: Proteomic Approach during the Induction of Somatic Embryogenesis in Coffea canephora
Source: Plants (Basel). 2023 Dec 7;12(24):4095. doi: 10.3390/plants12244095 (PMC10748034; doi:10.3390/plants12244095)
Supplement: Supplementary file 1 [file plants-12-04095-s001.zip › Table S1.pdf]

Table S1. Description of protein-protein interaction network of SE-related proteins.

Node	Domain summary
ABCB1	ABC transporter B family member 1: Auxin efflux transporter that acts as a negative regulator of light signaling to promote hypocotyl elongation. This transporter mediates the accumulation of chlorophyll and anthocyanin and the expression of genes in response to light. Participates directly in auxin efflux and thus regulates the polar (presumably basipetal) auxin transport (from root tips to root elongating zone). Transports also some auxin metabolites such as oxindoleacetic acid and indole acetaldehyde. It involves diverse auxin-mediated responses, including gravitropism, phototropism, etc.
ABCB21	ABC transporter B family member 21.
ABCB28	ABC transporter B family member 28 belongs to the ABC transporter superfamily. ABCB family. Multidrug resistance exporter (TC 3.A.1.201) subfamily.
ABCC1	ABC transporter C family member 1; Pump for glutathione S-conjugates. Mediates the transport of S-(2,4-dinitrophenyl)-glutathione (DNP-GS), GSSG, cyanidin 3-glucoside- GS (C3G-GS), and metolachlor-GS (MOC-GS); Belongs to the ABC transporter superfamily. ABCC family. Conjugate transporter (TC 3.A.1.208) subfamily.
ABCC14	ABC transporter C family member 14; Pump for glutathione S-conjugates; Belongs to the ABC transporter superfamily. ABCC family. Conjugate transporter (TC 3.A.1.208) subfamily.
ABCC2	ABC transporter C family member 2; Pump for glutathione S-conjugates. Mediates the transport of S-conjugates such as GSH, S-(2,4-dinitrophenyl)-glutathione (DNP-GS), GSSG, cyanidin 3-glucoside-GS (C3G-GS) and metolachlor-GS (MOC-GS), glucuronides such as 17-beta-estradiol 17-(beta-D-glucuronide) (E(2)17betaG), and of the chlorophyll catabolite such as B.napus nonfluorescent chlorophyll catabolite (Bn-NCC-1). This protein belongs to the ABC transporter superfamily. ABCC family. Conjugate transporter (TC 3.A.1.208) subfamily.
ABCC5	ABC transporter C family member 5; Pump for glutathione S-conjugates. It regulates K(+) and Na(+) cell content. Mediates resistance to NaCl and Li(+), confers sensitivity to sulfonylurea drugs such as glibenclamide (inducer of stomatal opening) and is required for stomatal opening regulation by auxin, abscisic acid (ABA), and external Ca(2+). Transports oestradiol-17-(beta-D-glucuronide) (E(2)17G). Involved in the root auxin content regulation that controls the transition from primary root elongation to lateral root formation. Plays a role in ABA-mediated germination inhibit [...]
ABCC8	ABC transporter C family member 8; Pump for glutathione S-conjugates.
ABCD1	ABC transporter D family member 1: Contributes to transporting fatty acids and their derivatives (acyl CoAs) across the peroxisomal membrane. Provides acetate to the glyoxylate cycle in developing seedlings. It is involved in pollen tube elongation, ovule fertilization, and seed germination after imbibition (controls the switch between the opposing developmental programs of dormancy and germination), probably by promoting beta-oxidation of storage lipids during gluconeogenesis. It is required to synthesize jasmonic acid and convert indole butyric acid to indole acetic acid.
ABCF1	ABC transporter F family member 1 belongs to the ABC transporter superfamily. ABCF family. EF3 (TC 3.A.1.121) subfamily.
ABCF3	ABC transporter F family member 3 belongs to the ABC transporter superfamily. ABCF family. EF3 (TC 3.A.1.121) subfamily.

ABCG1 ABC transporter G family member 1.

ABCG40 ABC transporter G family member 40; Maybe a general defense protein (By similarity). Functions as a pump to exclude Pb(2+) ions and/or Pb(2+)-containing toxic compounds from the cytoplasm. This transporter contributes to Pb(2+) ions resistance. Confers some resistance to the terpene sclareol.

ABCG7 ABC transporter G family member 7 belongs to the ABC transporter superfamily. ABCG family. Eye pigment precursor importer (TC 3.A.1.204) subfamily.

ABCI8 UPF0051 protein ABCI8, chloroplastic; involved in light signaling, probably by mediating the transport and correct distribution of protoporphyrin IX, a chlorophyll precursor, in response to far-red light; Belongs to the UPF0051 (ycf24) family.

AG Floral homeotic protein AGAMOUS: Probable transcription factor involved in the control of organ identity during the early development of flowers. It is required for normal development of stamens and carpels in the wild-type flower. It plays a role in maintaining the determinacy of the floral meristem. Acts as a C-class cadastral protein by repressing the A-class floral homeotic genes like APETALA1. Forms a heterodimer via the K-box domain with either SEPALATTA1/AGL2, SEPALATTA2/AGL4, SEPALLATA3/AGL9 or AGL6 that could be involved in genes regulation during floral meristem development.

APT1 Adenine phosphoribosyltransferase 1, chloroplastic: Catalyzes a salvage reaction resulting in the formation of AMP that is energetically less costly than de novo synthesis. It contributes primarily to the recycling of adenine into adenylate nucleotides but is also involved in the inactivation of cytokinins by phosphoribosylation. This enzyme catalyzes the conversion of cytokinins from free bases (active form) to the corresponding nucleotides (inactive form). This protein belongs to the purine/pyrimidine phosphoribosyltransferase family.

ARR14 Two-component response regulator ARR14; Transcriptional activator that binds specifically to the DNA sequence 5'-[AG]GATT-3'. Functions as a response regulator involved in the His-to-Asp phosphorelay signal transduction system. Phosphorylation of the Asp residue in the receiver domain activates the ability of the protein to promote the transcription of target genes. Could directly activate some type-A response regulators in response to cytokinins (By similarity); Belongs to the ARR family—Type-B subfamily.

ASK11 SKP1-like protein 11; Involved in ubiquitination and subsequent proteasomal degradation of target proteins. Together with CUL1, RBX1, and a F-box protein, it forms a SCF E3 ubiquitin ligase complex. The functional specificity of this complex depends on the type of F-box protein. The SCF complex serves as an adapter that links the F-box protein to CUL1 (By similarity). This protein plays a role during early flower reproductive development.

B''ALPHA Serine/threonine protein phosphatase 2A regulatory subunit B''alpha; Regulatory subunit of type 2A protein phosphatase. It is not involved in HMGR regulation in seedlings grown in standard medium but negatively regulates root growth in response to salt.

B''BETA Serine/threonine protein phosphatase 2A regulatory subunit B''beta; Regulatory subunit of type 2A protein phosphatase. Involved in post-transcriptional regulation of HMGR but not root growth regulation in response to salt.

B''DELTA Probable serine/threonine protein phosphatase 2A regulatory subunit B''delta; Probable regulatory subunit of type 2A protein phosphatase.

BIG Auxin transport protein BIG. This transporter is required for auxin efflux and polar auxin transport (PAT) influencing auxin-mediated developmental responses (e.g., cell

elongation, apical dominance, lateral root production, inflorescence architecture, general growth, and development). Controls the elongation of the pedicels and stem internodes through auxin action. Involved in the expression modulation of light-regulated genes. Represses CAB1 and CAB3 gene expression in etiolated seedlings.

CRT1 Calreticulin-1: Molecular calcium-binding chaperone promoting folding, oligomeric assembly, and quality control in the ER via the calreticulin/calnexin cycle. This lectin may interact transiently with almost all monoglucosylated glycoproteins synthesized in the ER (By similarity).

CRT2 Calreticulin-2: Molecular calcium-binding chaperone promoting folding, oligomeric assembly, and quality control in the ER via the calreticulin/calnexin cycle. This lectin may interact transiently with almost all monoglucosylated glycoproteins synthesized in the ER (By similarity).

DRMH1 Dormancy-associated protein homolog 1.

GRF1-2 14-3-3-like protein GF14 chi is associated with a DNA binding complex that binds to the G box, a well-characterized cis-acting DNA regulatory element in plant genes that regulates nutrient metabolism.

GRF11 14-3-3-like protein GF14 omicron is associated with a DNA binding complex that binds to the G box, a well-characterized cis-acting DNA regulatory element found in plant genes.

GRF12 14-3-3-like protein GF14 iota is associated with a DNA binding complex that binds to the G box, a well-characterized cis-acting DNA regulatory element in plant genes.

GRF2 14-3-3-like protein GF14 omega is associated with a DNA binding complex that binds to the G box, a well-characterized cis-acting DNA regulatory element found in plant genes. This protein belongs to the 14-3-3 family.

GRF3 14-3-3-like protein GF14 psi is associated with a DNA binding complex that binds to the G box, a well-characterized cis-acting DNA regulatory element found in plant genes that regulates nutrient metabolism—reciprocal negative transcription regulation of miR396. Negative regulator of constitutive freezing tolerance and cold acclimation by controlling cold-induced gene expression partially through ethylene (ET)-dependent pathway; prevents ethylene (ET) biosynthesis, probably by binding 1- aminocyclopropane-1-carboxylate synthases (ACS) to reduce their stability.

GRF4 14-3-3-like protein GF14 phi is associated with a DNA binding complex that binds to the G box, a well-characterized cis-acting DNA regulatory element in plant genes.

GRF5 14-3-3-like protein GF14 upsilon is associated with a DNA binding complex that binds to the G box, a well-characterized cis-acting DNA regulatory element found in plant genes. It may be involved in cell cycle regulation by binding to soluble EDE1 and sequestering it in an inactive form during the early stages of mitosis.

GRF7 14-3-3-like protein GF14 nu is associated with a DNA binding complex that binds to the G box, a well-characterized cis-acting DNA regulatory element found in plant genes.

GRF8 14-3-3-like protein GF14 kappa is associated with a DNA binding complex that binds to the G box, a well-characterized cis-acting DNA regulatory element in plant genes that regulates nutrient metabolism. Negative regulator of freezing tolerance that modulates cold-responsive C-repeat-binding factors (CBF) DREB1A AND DREB1B proteins stability by facilitating their ubiquitin-mediated degradation.

GRF9-2 14-3-3-like protein GF14 mu is associated with a DNA binding complex that binds to the G box, a well-characterized cis-acting DNA regulatory element found in plant genes. This protein belongs to the 14-3-3 family.

ILR1 IAA-amino acid hydrolase ILR1 hydrolyzes specific amino acid conjugates of the plant growth regulator indole-3-acetic acid (IAA), including IAA-Phe, IAA-Leu, and IAA-Tyr. We

can also use IAA-Ala, IAA-Gly, IAA-Met, and IAA-Glu as substrates with low efficiency. This enzyme does not show activity with IAA-Ile, IAA-1-O-beta-D-glucose, or IAA-myo-inositol. It is the most efficient enzyme of the ILL family for IAA-Leu hydrolysis. Necessary for IAA-Leu and IAA-Phe hydrolysis in roots. May act with ILL2 to provide free IAA to germinating seedlings. This hydrolase belongs to the peptidase M20 family.

LOG3	Cytokinin riboside 5'-monophosphate phosphoribohydrolase LOG3. Cytokinin-activating enzyme working in the direct activation pathway. Phosphoribohydrolase converts inactive cytokinin nucleotides to biologically active free-base forms.
NFYB1	Nuclear transcription factor Y subunit B-1. Component of the NF-Y/HAP transcription factor complex. The NF-Y complex stimulates the transcription of various genes by recognizing and binding to a CCAAT motif in promoters belonging to the NFYB/HAP3 subunit family.
NFYB3	Nuclear transcription factor Y subunit B-3. Component of the NF-Y/HAP transcription factor complex. The NF-Y complex stimulates the transcription of various genes by recognizing and binding to a CCAAT motif in promoters.
NFYC4	Nuclear transcription factor Y subunit C-4 stimulates the transcription of various genes by recognizing and binding to a CCAAT motif in promoters (By similarity). It is involved in the abscisic acid (ABA) signaling pathway.
PP2A2-2	Serine/threonine-protein phosphatase PP2A-2 catalytic subunit: Dephosphorylates and activates the actin-depolymerizing factor ADF1, which, in turn, regulates actin cytoskeleton remodeling and is involved in the blue light photoreceptor PHOT2-mediated chloroplast avoidance movements. This protein is associated with the serine/threonine-protein phosphatase PP2A regulatory subunits A and B' to positively regulate the beta-oxidation of fatty acids and protoauxins in peroxisomes by dephosphorylating peroxisomal beta-oxidation-related proteins. It acts as a negative regulator of abscisic acid (ABA) signaling.
PP2A4	Serine/threonine-protein phosphatase PP2A-4 catalytic subunit; Functions redundantly with PP2A3 and is involved in establishing auxin gradients, apical-basal axis of polarity, and root and shoot apical meristem during embryogenesis. May dephosphorylate PIN1 and regulate its subcellular distribution for polar auxin transport. The holoenzyme composed of PP2AA1, PP2A4, and B'ZETA or B'ETA acts as a negative regulator of plant innate immunity by controlling the BAK1 phosphorylation state and activating surface-localized immune receptor complexes. This phosphatase belongs to the PPP phosphatase family.
PP2AA1	Serine/threonine-protein phosphatase 2A 65 kDa regulatory subunit A alpha isoform; The A subunit of protein phosphatase 2A serves as a scaffolding molecule to coordinate the assembly of the catalytic subunit and a variable regulatory B subunit. It seems to act as a positive regulator of PP2A catalytic activity. This protein confers resistance to phosphatase inhibitors such as okadaic acid and cantharidin. It is involved during developmental processes such as seedling and floral development, root gravitropism, and stomatal opening regulation and regulating auxin efflux.
PP2AA2	Serine/threonine-protein phosphatase 2A 65 kDa regulatory subunit A beta isoform; The A subunit of protein phosphatase 2A serves as a scaffolding molecule to coordinate the assembly of the catalytic subunit and a variable regulatory B subunit. It is involved during developmental processes such as seedling and floral development. It seems to act as a negative regulator of PP2A catalytic activity. This protein associates with the serine/threonine-protein phosphatase PP2A catalytic subunit C and regulatory subunit B' to positively regulate the beta-oxidation of fatty acids and protoauxins in

	peroxisomes.
PP2AA3	Serine/threonine-protein phosphatase 2A 65 kDa, regulatory subunit A gamma isoform. The A subunit of protein phosphatase 2A is a scaffolding molecule to coordinate the assembly of the catalytic subunit and a variable regulatory B subunit. It is involved in developmental processes such as seedling and floral development. It seems to act as a negative regulator of PP2A catalytic activity.
PP2AB2	Serine/threonine protein phosphatase 2A 55 kDa regulatory subunit B beta isoform; The B regulatory subunit may modulate substrate selectivity and catalytic activity and also may direct the localization of the catalytic enzyme to a particular subcellular compartment.
SERK1	Somatic embryogenesis receptor kinase 1: Dual specificity kinase acting on serine/threonine- and tyrosine-containing substrates. Phosphorylates BRI1 on 'Ser-887' and CDC48 on at least one threonine residue and on 'Ser-41'. This protein confers embryogenic competence. This receptor acts redundantly with SERK2 as a control point for sporophytic development, controlling male gametophyte production. It is involved in the brassinolide signaling pathway.
TSB2	Tryptophan synthase beta chain 2, chloroplastic. The beta subunit synthesizes L-tryptophan from indole and L-serine and belongs to the TrpB family.
